# Prevalence of Poor Diabetes Self-Management Behaviors among Ethiopian Diabetes Mellitus Patients: A Systematic Review and Meta-Analysis

**DOI:** 10.4314/ejhs.v30i4.18

**Published:** 2020-07-01

**Authors:** Teshome Tesfaye Habebo, Ebrahim Jaafari Pooyan, Ali Mohammad Mosadeghrad, Getachew Ossabo Babore, Blen Kassahun Dessu

**Affiliations:** 1 Tehran University of Medical Sciences, international campus (TUMS-IC), Tehran, Iran; 2 School of Public Health, Tehran University of Medical Sciences (TUMS), Tehran, Iran; 3 Kembata Tembaro zone Health department, SNNPRS, Ethiopia; 4 Department of nursing, college of medicine and health sciences, Wachemo University, Hosanna, Ethiopia; 5 Department of anesthesia, college of health sciences and medicine, Wolaita Sodo University, Wolaita Sodo, Ethiopia

**Keywords:** Diabetes mellitus, Ethiopia, Meta-analysis, poor self-management, Prevalence, Systematic review

## Abstract

**Background:**

Diabetes has no cure so far, but appropriate self-management contributes to delay or control its progression. However, poor self-management by diabetic patients adds to disease burden. The pooled prevalence of overall, and its main components of poor self-management among Ethiopian diabetic patients remain elusive. Hence, this study aimed to determine the prevalence of poor diabetes self-management behaviors among diabetic patients in Ethiopia.

**Method:**

by using different combinations of search terms, we accessed articles done until February 15, 2020 through Pubmed, Scopus, Web of Science and Embase databases. Newcastle-Ottawa quality assessment scale was used for quality assessment, and STATA version 14 software along with the random-effects model was employed for statistical analyses. The Preferred Reporting Items for Systematic Reviews and Meta-Analyses (PRISMA.) guideline was followed to report the results.

**Result:**

Twenty-one studies with 7,168 participants were included in this meta-analysis. The overall pooled prevalence of poor self-management behavior among diabetic patients in Ethiopia was 49.79% (95% CI: 43.58%, 56.01%). Based on subgroup analysis, the estimated magnitudes of poor self-management by regions were 68.58% in Tigray, 55.46% in Harari, 54.74%, in Amhara, 40.90%, in SNNPRS and 37.06% in Addis Ababa. The worst (80.91%) and relatively better (24.65%) self-management components were observed on self-blood glucose monitoring and medication adherence, respectively.

**Conclusion:**

One in two diabetic patients in Ethiopia had poor self-management. Thus, we strongly recommend to the ministry of health and universities to train diabetes health educators, and the health facilities to deliver tailored diabetes health education.

## Introduction

Non-communicable diseases (NCDs) such as cardiovascular diseases, cancer, stroke, and diabetes mellitus (DM.) appear to be the main health threats to human beings throughout the world (1). Fast socio-demographic and epidemiological transitions catalyzed by risky lifestyles in developing countries contribute the highest share to NCDs (2). Avoidable behavioral risk factors like unhealthy eating habits, physical inactivity, tobacco, and alcohol consumption are the main contributing factors for both disease occurrences and its progression (3).

Diabetes mellitus, one of the four major NCDs, is a metabolic disorder of multiple etiologies and characterized by abnormally elevated blood glucose levels due to carbohydrate, fat and protein metabolism disturbances. DM originates from defects in insulin secretion, insulin action, or both (4). Type 1, type 2, and gestational diabetes are the three main categories of diabetes mellitus (5). These days, 1 in every 11 individuals is diabetic, half of the estimated 451 million people living with diabetes are undiagnosed whereas 5 million deaths are attributable to diabetes occurrence every year across the world (6).

Thus, diabetes has huge economic, social and health consequences; and the burden has been dual and very significant in resources limited settings and countries (7). For instance, global healthcare expenditure for diabetes management was US$ 850 billion in 2017 (6). Unproportionally, Sub-Saharan Africa (SSA) contributes the heaviest global burden of DM; the region continues to host the big share (8). The second most populated African country, Ethiopia, is also one of these countries where the trend of diabetes mellitus burden is alarmingly increasing (3, 7).

Although there is no cure for DM so far, prevention, delay and attaining better health status have been possible (5). These could not be only due to the advancements in medicine and technologies but mainly by shifting emphasis from disease treatment to person-oriented approaches and preventive measures. Nonetheless, the achievements are far below satisfactory in Ethiopia (9). Additionally, the lack of access to quality healthcare and weak preventive measures have been contributing to impoverishment and premature deaths in the country (10). However, attaining a better clinical outcome is possible when diabetic patients actively manage and stick to healthy lifestyles (5).

Besides, diabetes management goals can be attained to the better possible levels even without more advanced technologies and/or medicines (11). This could be achieved by appropriate diabetes self-management measures such as self-monitoring of blood glucose (SMBG), dietary management, regular physical exercise, good adherence to medications and foot care by diabetic patients themselves (12).

Therefore, diabetes management goals could be achieved precisely by engaging the patients, delivering comprehensive care and by patient-centered goal setting. For instance, maintaining optimum glycaemic level, a cornerstone of diabetes management, reduces diabetes-related complications, rate of admissions and premature deaths (13). On the other hand, each diabetic individual needs a care plan and systemic approaches to attain a set goal rather than treating only his or her illnesses (8). Thus, empowering individuals to make effective decisions on their health and to become crucial role players on their health rather than only collecting and taking their refilling medicines is critically important to improving the status of the patients (14).

Some studies indicated that until these days, most of these self-management components have been underestimated and poorly understood in Ethiopia. Therefore, we planned and conducted this systematic review and meta-analysis to determine the pooled prevalence of overall, and its main components of poor self-management behaviors among diabetic patients in Ethiopia that may play a crucial role to develop and implement appropriate policies to deal with the problem.

## Methods And Materials

### Search strategy

A systematic review and metaanalysis was done on the prevalence of poor self-management behavior, and its main components among diabetic patients in Ethiopia. A comprehensive systematic search for all relevant studies was carried-out in Pub-med, Scopus, Embase, and Web of Science from inception to February 15, 2020 and additional supplementary search for articles on national websites, Google, and Google scholar and cross-reference searches were also undertaken. The Preferred Reporting Items for Systematic Reviews and Meta-Analyses PRISMA guideline (15) was applied to plan and carry out this systematic review and meta-analysis.

The search terms used include: “adherence” OR “Prevalenc*” OR “Compliance” OR “predict*” OR “Determin*” OR “Level” OR “Magnitude” OR “Effect” combined with “Diabetes mellitus” OR “Diabet*” OR “Diabetic patients” OR “Diabetes mellitus patients” and “self-management” OR "self-care behavior" OR “Self-care activities” OR “Self care practice” OR “self-care measures” OR "self-care actions” and “Ethiopia”. Boolean operators “AND” and “OR” were used to combine the search terms as appropriate.

During searching for articles, language or time limits were not applied. All relevant published and unpublished studies up to 15th February 2020 were included in this review, and the systematic literature searches were done from 1^st^ January to 15^th^ February 2020 by two independent researchers.

### The outcome of the study and operational definitions

The primary outcome of this study was estimating the national pooled prevalence of poor self-management behavior among DM patients in Ethiopia. **Self-management behaviors** are those activities that a diabetic patient initiates and performs on his or her own for controlling his or her disease, maintaining life, health and wellbeing which includes self-monitoring of blood glucose (SMBG), dietary management, physical exercise, adherence to medication, foot care, self-efficacy, and social support.

### Poor self-management

This means when the diabetic patient fails to perform at least an average score of the recommended self-management activity. Thus, in this article, we considered the mean scores as cut-off points as reported by each study. Our review question was “What is the pooled prevalence of overall poor self-management behavior, and its main components among diabetic patients in Ethiopia?”

### Study selection and eligibility criteria

This article incorporated all studies that were done on self-management behaviors among diabetic patients irrespective of types of diabetes mellitus, age, sex, and other characteristics. Articles were eligible when they got ethical approval, had a response rate of ≥85%, reported either overall or sufficient data to calculate it; studies from Ethiopia’s territories, and peer-reviewed studies.

Articles were considered for exclusion when they were not primary articles, did not present sufficient data to calculate the outcome of interest, or presented in more than one publication. Hence, all studies that were in the form of journal articles, master’s thesis, and dissertations were also considered for inclusion. After retrieval of articles from the electronic databases, screening was done step by step. First they were screened based on the title, by abstract and finally by full texts.

### The review process and quality appraisal

In this study, the quality of each article was assessed by applying a critical appraisal tool for use in the systematic review for prevalence studies (16). Two authors (TTH and GOB) independently identified, retrieved and checked all eligible articles step-wise based on titles, abstracts, and full texts, and the methodological quality appraisal was also done independently. Any inconsistencies were referred to a third independent referee (EJP) for discussion based on pieces of evidence and resolved objectively. The modified version of the Newcastle-Ottawa scale quality assessment framework for cross-sectional studies (NOS) was used to assess the quality of the included studies (17).

### Data extraction

After searching for all relevant studies in predetermined search sources, Endnote X7 reference management software for windows (Thomson Reuters, USA) was used to download, organize, review, and cite the articles. We developed Microsoft Excel data extraction form, piloted and used to record relevant information such as author name, year of publication, geographic location, sample size, sex, study design, DM types, response rate, lowest age included, mean age, overall poor self-management proportions/numbers and other dimensions of diabetics’ self-management.

### Synthesis and analysis

After extracting the data from all eligible studies, the data were entered into STATA software for windows (version 14) and the analysis was done. The random-effects model was used for estimating the pooled prevalence of overall poor self-management behaviors and its main components. During meta-analysis, one of the relevant statistical issues is managing the heterogeneity among studies. To handle this, a commonly recommended method was applying the random-effects model, specifically DerSimonian and Laird method, in the metaanalysis because it assumes heterogeneity across studies (18, 19). Thus, we applied this method and all data processing and statistical analyses were carried out by using STATA version 14 (STATA Corporation, College Station, Texas, USA).

I^2^ test was used to check the heterogeneity of included studies. This statistical method, which ranges between 0% and 100%, is applied to quantify the percentage of the total variation in study estimate due to heterogeneity (20) and a P-value of less than 0.05 was considered to indicate the presence of heterogeneity. In this paper, the values of I^2^ were very high (≥75%) which implied high heterogeneity so that the random effect model at 95% confidence level (CI) was used to adjust for the observed variability.

Additionally, the existence/source of heterogeneity was explored by subgroup analysis and meta-regression. Funnel pilot visualization was done to investigate possible publication bias. Begg’s and Egger’s tests were applied to investigate the possible publication bias and less than 0.05 P-value was considered to determine the statistical significance of publication bias as suggested by scholars (21). Furthermore, to investigate the potential influence of individual studies on pooled estimates, a sensitivity analysis was also carried out and presented in the following section.

## Results

### Study selection

**Figure1:**
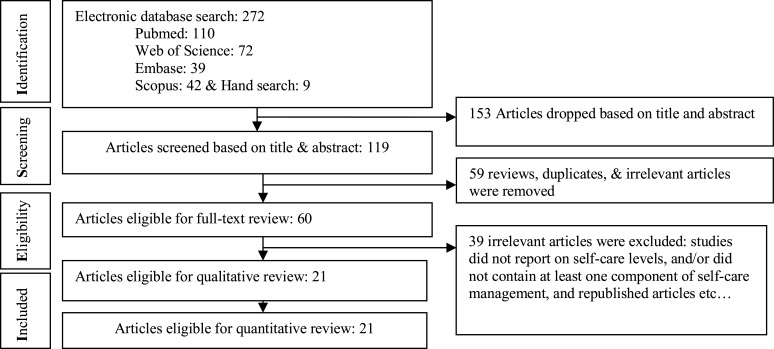
The process of systematic literature search and screening flow diagram on the prevalence of poor self-management behaviors among diabetic patients in Ethiopia, 2020

The search strategy identified a total of 272 articles. On the first step, 153 irrelevant documents were removed, and then we screened the remaining articles based on titles and abstracts. After screening of title and abstract, full texts according to the eligibility criteria, 21 articles met the inclusion criteria and are included in this systematic review and metaanalysis. Although we did not apply language limits while searching for articles, there was no article retrieved published in other than the English language. The process of systematic literature retrieval and screening of the papers are presented in Figure 1.

### Patients and characteristics of the included studies

Totally, 21 studies were eligible and included in this study. All the included studies were cross-sectional by design, and 7168 patients participated. Thirteen of the studies were done by recruiting both types I and II diabetic patients while eight were conducted by recruiting only type II diabetic patients. The sample size varied from 194(22) to 637(23), the minimum response rate of the included studies was 95 percent(24, 25) and the maximum was 100%. Two studies included patients with the youngest age of 15 years(22, 26), seventeen studies recruited 18 years and older, others started from older than 25 years (27), and 30 years (28).

Regarding the geographic locations of included studies, five were conducted in Oromia, three in Addis Ababa, two in Tigray, five in Amhara, three in Harari and Dire Dawa, two in SNNPRS and only one study was conducted in Benishangul Gumuz but we could not find any study from Afar, Gambella, and Somali regions. Most of the studies were conducted in a single health facility, and the mean age of included studies ranged from 38 to

55.2 years. The overall quality of the included studies was good and ranged from 5 to 8 against the NOS scale and the results are presented in Table 1.

**Table 1:** Characteristics of included studies in systematic review and meta-analysis on the prevalence of poor self-management behaviors among diabetic patients in Ethiopia, 2020

Author [reff.]	Pub. year	study region	Sample	females No	Study design	included DM type	Response rate (%)	lowest age	mean age	NOS score	Prevalence (95% CI)
Abate et al (29)	2018	Amhara	416	176	cross sectional	I & II	99.5	18	41.1	8	71.63(67.30,75.97)
Amente et al (26)	2014	Oromia	254	119	cross sectional	I & II	98	15	38	7	45.28(39.15,51.3)
Aschalew et al (30)	2019	Amhara	403	183	cross sectional	I & II	100	18	NS	8	51.86(46.98,56.74)
Ayele et al (31)	2019	Harari	320	178	cross sectional	I & II	97.8	18	51	8	61.88(56.55,67.20)
Ayele K et al (32)	2012	Harari	222	134	cross sectional	I & II	100	18	49.7	7	60.81(54.39,67.23)
Chali et al (33)	2018	B.Gumuz	383	174	cross sectional	I & II	96	18	44.5	7	45.69(40.70,50.68)
Dedefo et al (34)	2019	Oromia	252	114	cross sectional	I & II	100	18	41.7	7	39.29(33.26,45.32)
Feleke et al (35)	2013	Amhara	410	212	cross sectional	I & II	97.2	18	41.9	8	63.17(58.50,67.84)
Asmare et al (36)	2018	D/Dawa	506	279	cross sectional	I & II	98.6	18	51.48	7	44.07(39.75, 48.40)
Gurmu et al (37)	2018	Oromia	257	118	cross sectional	II	100	18	42.9	7	45.53(39.44,651.61)
Kassahun et al(24)	2016	Oromia	309	120	cross sectional	II	95	18	NS	7	50.81(45.23, 56.38)
Mariye et al (38)	2018	Tigray	284	NS	cross sectional	II	100	18	52.19	7	62.68(57.05,68.30)
Melat et al (23)	2016	AA	637	347	cross sectional	I & II	97.8	18		5	39.72(35.92,43.52)
Niguse et al (39)	2019	Tigray	338	154	cross sectional	I & II	100	18	45.8	8	74.26(69.60,78.92)
Sorato et al (22)	2016	SNNPRS	194	99	cross sectional	II	100	15	50.3	7	58.76(51.84, 65.69)
Addisu et al (40)	2014	SNNPRS	310	110	cross sectional	I & II	100	18	41.9	8	23.23(18.53,27.93)
Feyissa et al (41)	2014	AA	324	174	cross sectional	II	98.8	18	52.8	7	48.46(43.02,53.90)
Berhe KK et al (27)	2014	Tigray	300	127	cross sectional	II	96.8	25	50.02	7	49.00(43.34,54.66)
Tiruneh et al (25)	2019	Amhara	385	183	cross sectional	II	95	18	52.28	8	36.88(32.06,41.70)
Fikadu et al (42)	2017	Amhara	344	NS	cross sectional	I & II	98	18	NS	7	50.00(44.72,55.28)
Berhe et al (28)	2012	AA	320	153	cross sectional	II	99.1	30	55.03	7	23.13(18.51,27.74)

NS=not specified, SNNPRS= south nations nationalities & people regional state, AA= Addis Ababa, DM=diabetes mellitus

### The magnitude of poor self-management activities (meta-analysis)

**Figure 2:**
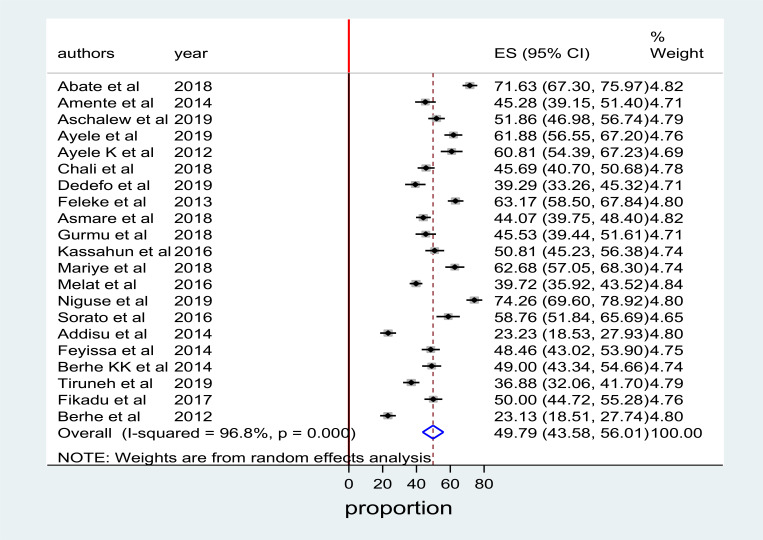
forest plot depicting the prevalence of poor self-management among DM patients in Ethiopia, 2020.

The pooled magnitude using the fixed-effect model indicated significant heterogeneity between studies. Hence, we performed the analysis by applying the random-effects model. By using the random-effects model, the overall pooled prevalence of poor self-management among diabetes mellitus patients in Ethiopia was 49.79% (95% CI: 43.58%, 56.01%) with significant heterogeneity between studies (I^2^ = 96.80%, P=0.001). The overall pooled prevalence of poor self-management analysis is presented using a forest plot in Figure 2.

**Figure 3:**
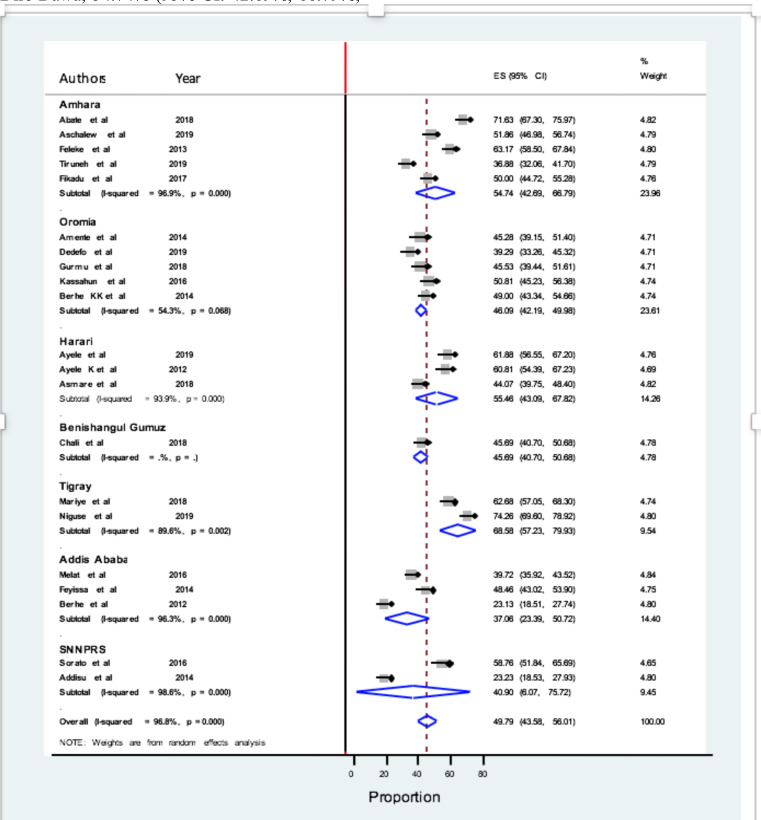
Prevalence of poor self-management behaviors among DM patients by region in Ethiopia, 2020.

A subgroup analysis by region was done to investigate the possible heterogeneity between studies. Of the 21 included studies, the highest pooled magnitude of poor self-management was 68.58% (95% CI: 57.23%, 79.93%; I^2^ = 89.6%) in Tigray, followed by 55.46% (95% CI: 43.09%, 67.82%; I^2^ = 93.9%) in Harari and I^2^ = 96.9%) in Amhara, 40.90% (95% CI: 6.07%, 75.72%; I^2^ = 98.6%) in SNNPRS and 37.06% (95% CI: 23.39%, 50.72%; I^2^ = 96.3%) in Addis Ababa, respectively. It was not significant in other regions like Oromia, 46.09% (95% CI: 42.19%, 49.98%; I^2^= 54.03%) (Figure 3).

### Heterogeneity investigation

Due to the differences in quality of studies, methodology, sample sizes, and inclusion criteria in each study, heterogeneity in systematic review and meta-analysis is inevitable. In our work, the I^2^ values revealed heterogeneity as well. Hence, we applied the random effect model for analysis to adjust the observed variabilities. Additionally, the possible heterogeneity existence was investigated by subgroup analysis.

Nonetheless, the magnitude of heterogeneity was lower after subgroup analysis. Though, this was the case, as meta-regression is commonly suggested to investigate heterogeneity because it has the potential benefit of letting the investigation of single or more covariate at the same time (35). Thus, we further conducted heterogeneity investigation with the metaregression model by introducing the publication year and sample size as covariates. However, the meta-regression result indicated that there was no statistically significant heterogeneity for both covariates.

### Publication bias

**Figure 4:**
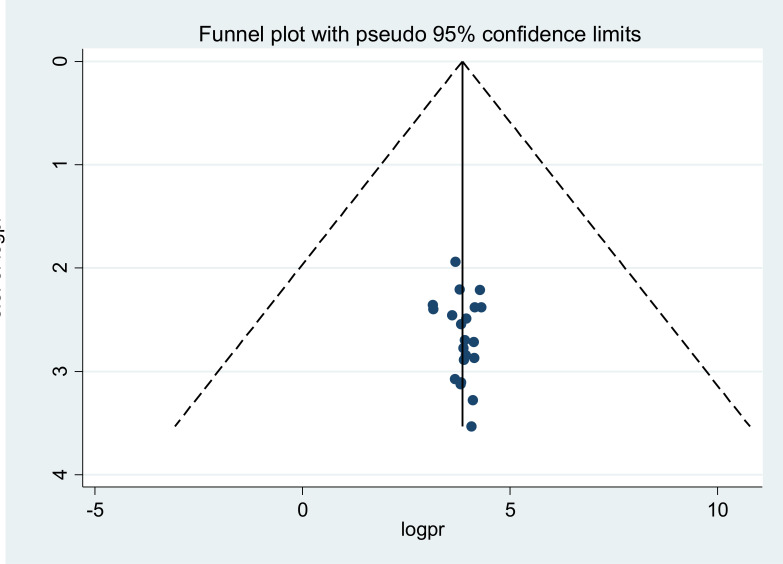
Funnel plots depicting publication bias test of the 21 studies on prevalence of poor self-management among DM patients in Ethiopia, 2020.

To assess the presence of publication bias, using different methods that single-out the sources and its magnitude has been recommended by scientist (36). To do this, funnel plots and tests like egger and Begg are being suggested commonly. In funnel plots, each point corresponds to each study while the asymmetrical distribution of the studies depicts the presence of publication bias. Therefore, by using funnel plots, and Begg’s and egger’s tests we investigated the presence of publication bias. However, in our meta-analysis, the funnel plots and tests (P-values > 0.05) implied no strong evidence for the presence of publication bias (Figure 4).

### Sensitivity analysis

**Figure 5:**
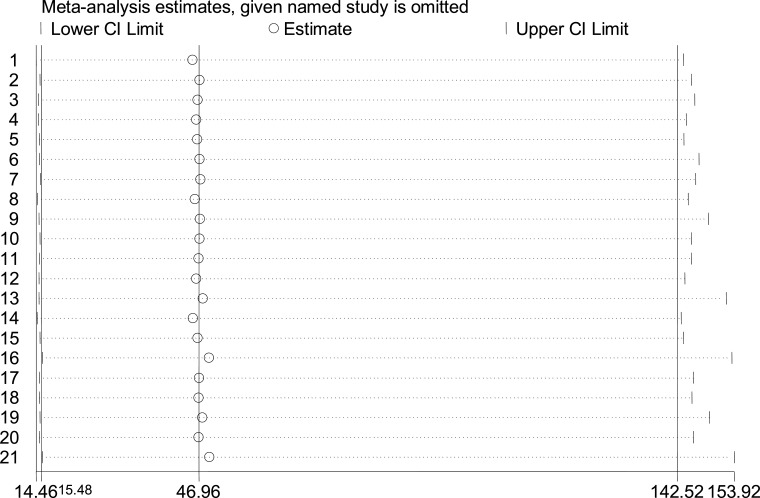
Sensitivity analysis result of the 21 included studies in meta-analysis, 2020

To explore the potential effect of missing data and the influence of a single study on the overall estimate, doing sensitivity analysis is critically important. Thus, we did sensitivity analysis with a random-effects model but the result showed that no single study unduly influenced the overall magnitude estimate of poor self-management activities among diabetic patients in Ethiopia (Figure 5).

### Contributors for poor self-management

Studies reported different positively associated factors of diabetics' poor adherence to self-management, and we categorized as community-related factors such as lack of family and social support(24, 37, 38), lower education levels (23, 24), and living in rural areas (22, 23, 39). Health system-related factors like lack of diabetic health education (24, 40) and low treatment satisfaction level (40). Patient-related factors such as lack of access to SMBG and not having glucometer (24) (41), poor diabetes-related knowledge (24, 32, 41), low self-efficacy (24), lower socio-economic status (23, 32) and disease-related factors such as less duration since diagnosis (32, 34), presence of diabetes-related complications and co-morbidities (23, 41). However, some studies reported conflicting findings such as high economic status (40), more than 10 years duration with the disease (40), having strong social support and good diabetes-related knowledge (23) as positive predictors of poor diabetes self-management.

### Diabetes self-management components

Diabetes self-management has many dimensions; for instance, some of the scholars classify this into eight (27) such as diet, exercise, self-blood glucose testing, foot-care, medication adherence, diabetes knowledge, self-efficacy, and social support whereas others considered only five of these dimensions (22, 40). Though the dimensions are many and encompass various aspects, the intent of all has been to improve the health status of the diabetic people and to minimize the negative health consequences on the patients, families, and health systems at large; we presented the main components as follows.

**i. Prevalence of poor foot care-**The overall pooled prevalence of poor foot care was 34.76% (95%CI: 23.12, 46.39; I^2^=97%, P=0.001) among diabetic patients in Ethiopia. Only five (23, 27, 34, 36, 41) out of the 21 studies included in this meta-analysis reported the magnitude of poor foot care component by involving a total of 2019 patients of which 684 of had poor foot care practices. Despite the mere importance of the foot care practices by diabetic patients to avoid or decrease the foot ulcers and amputation on worse occasions, our finding shows that the foot care activities by the Ethiopian diabetic patients has been less researched and ignored component.
**ii. Prevalence of poor dietary management-**Dietary management is one of the cornerstones for DM patients to improve their health status and maintain healthy conditions so that all of the diabetic patients are expected to stick to it. Seven (22, 28, 32, 34, 35, 40, 41) of the 21 studies investigated the level of diabetic patients’ dietary management component. One thousand twenty-four out of two thousand thirty-two included patients had poor dietary management practice as one of their self-diabetes management components. Thus, the estimated pooled prevalence of poor dietary management among diabetics in Ethiopia became 49.22% (95% CI: 36.33, 62.11; I^2^=97.4%, P=0.001). This implies that more than half of the Ethiopian diabetics do not adhere to the food recommendations.
**iii. Prevalence of poor SMBGS-**elfmonitoring of blood glucose is one of the critically important dimensions that diabetic patients are expected to base on their health conditions and it has been suggested to them to have their glucometer as well. Regarding this, ten out of the twenty-one included studies explored its level. Of the 3475 patients who had participated in ten studies, 2733 patients had poor adherence to it. Thus, the pooled prevalence of poor SMBG behavior was 80.91% (95% CI: 75.14%, 86.68%; I^2^=95.3%, P=0.001). This shows almost negligible numbers of the patients were doing SBMG. Hence, it needs more effort to change the scenario.
**iv. Prevalence of poor adherence to physical exercise-**Regular physical exercise is one of the very important factors that have a potential impact to improve the health of people, in our case the DM patients, and it contributes to making diabetic care more effective. Therefore, the world health organization (WHO) and scholars recommend regular physical activities for DM patients. However, it needs good monitoring and attention from health educators. Eight of the included studies by involving 2745 patients investigated this component and reported that 1441 patients had poor adherence to the recommended physical exercise in Ethiopia. Thus, it yielded the pooled prevalence of poor adherence to physical exercise of 50.45% (95% CI: 37.81%, 63.10%; I^2^= 98%, P=0.001).
**v. Prevalence of poor adherence to prescribed medication-**All diabetic patients who take medications for their diabetes treatment should adhere to it so that they could control the glycemic level, reduce the occurrence of complications, and live healthily. Eight of the studies by interviewing 3003 diabetic patients studied this component and the overall pooled prevalence of poor adherence to prescribed medication was 24.65% (95% CI: 12.66%, 36.65%; I^2^= 99.2%, P=0.001). The studies were conducted in Amhara (30), Oromia (24), Addis Ababa (23, 28, 41), Dire-Dawa (36) and SNNPRS (22, 40) regions whereas the highest and the lowest prevalence were from Oromia Region (24) and Addis Ababa City Administration (28), respectively.
**vi. The magnitude of poor social support-**As presented by scholars, social support and self-efficacy could enhance self-confidence in diabetes self-management activities, glycemia control, and ultimately health status. Therefore, social support is a fundamental approach to sustaining self-management behaviors and overcoming barriers among patients with DM. Seven of the twenty-one included studies by involving 2602 patients studied this component. The studies were conducted in four regions: three in Amhara (25, 30), two in Oromia (34, 37), single studies in Dire-Dawa (36) and Benishangul Gumuz (33). The pooled prevalence of poor social support for Ethiopian diabetic patients was 54.32% (95% CI: 34.70%, 73.93%; I^2^= 99.2%, P=0.001) in our analysis.

## Discussion

Although diabetes mellitus is none curable, prevention, delay and living healthy are possible (5). However, it needs diabetic patients' dedication to recommended self-management behaviors in multiple domains and delivering relevant health services by the healthcare systems (43, 44).

Thus, diabetes self-management behaviors are multidimensional and need multidisciplinary approaches as well as lifestyle modifications like smoking cessation and avoiding alcohol consumption by diabetic patients (45, 46). Despite their mere importance, most of these dimensions have been less monitored and poorly understood in Ethiopia.

The overall pooled prevalence of poor self-management behavior among diabetic patients in Ethiopia was 49.79%. Thus, our study demonstrated that the magnitude of poor self-management among diabetic patients in the country was very high which implies almost one of every two diabetic patients in Ethiopia does not adhere to the recommended self-management activities. This finding is worse compared to studies done in China (6%) (47) and in Iran (26.2%) (48) but better compared to a study conducted in India (75%) (49). The possible reason or this disagreement might be the difference in sample size and study settings.

Thus, high prevalence of poor self-management behaviors among diabetics in Ethiopia is a critical problem. This could poses huge burden both to the society as a whole and to the individual patient particularly by increasing the medical care cost on the one hand and this may indicate the poor healthcare services, specifically health education services, in the healthcare facilities on the other hand.

Therefore, unless appropriate intervention is put in place to avert this problem, poor self-management hampers the quality life of the diabetic people because most of the diabetes-related adverse health outcomes such as diabetes-related complications, morbidities and mortalities are the bi-products of poor self-management behaviors in one or another way (50).

Subgroup analysis was also done to estimate the prevalence by region. Due to geographical proximity and involvement of patients from both settings, we included one of the studies from Dire-Dawa into Harari for subgroup analysis. Based on this, the pooled estimates of poor self-management prevalence by region was (in descending order) 68.58% in Tigray, 55.46% in Harari, 54.74% in Amhara, 40.90% in SNNPRS, and 37.06% in Addis Ababa, whilst it was not statistically significant in Oromia region despite five studies were eligible and included from the region.

The regional difference might be due to the difference in socioeconomic status. For instance, high proportions of illiteracy level, and very low and low monthly income levels were reported in studies from the Tigray Region as compared to studies in Addis Ababa. Additionally, the difference between the highest and the lowest was almost twice (1.85*). On top of this, Addis Ababa is more urbanized than Tigray, and it has better access to services and information than others have. Hence, education, income, and residence might have influenced the self-management behaviors of the patients.

Additionally, we did meta-analysis for major self-management components to estimate the pooled prevalence of each component. The pooled prevalence of poor foot care behavior among Ethiopian diabetics was 34.76%. This figure is significantly lower than studies done in Iran (60%) (51) and in Nigeria (89.8%) (52). Sixty percent of the study participants in Iran failed to inspect their feet whereas nine in every ten patients in Kenya had poor foot care behaviors. Although our finding was relatively lower than the reports of few studies from elsewhere, one in every three diabetic individuals who had poor foot self-management practice in Ethiopia is one of the critical issues as compared to its relevance by reducing foot ulcers, peripheral vascular diseases, amputations, and other adverse consequences.

In our study, the pooled prevalence of poor dietary self-management behaviors among Ethiopian diabetics was 49.22%. This finding is comparable to a study finding in Egypt (58.3%) (53), but not from Nigeria (33.7%) (54). Nonetheless, the extent to which diabetic patients adhere to dietary recommendations depends on the involvement and guidance from a healthcare provider and contextualized approaches as well as practicing with a partner or in a group (55). Furthermore, in Ethiopia’s context, the recommended food availability, affordability, patients’ low level of understanding and neglect of health education in healthcare facilities might have been widening the gaps.

The current study also revealed that the pooled prevalence of poor SMBG was 80.91% among Ethiopian diabetics. This finding agrees with a study report from Egypt where 78.6% of the study participants did not adhere to self-blood glucose monitoring (53). However, this finding does not agree with findings from Saudi Arabia (29.2%) (56), Poland (40%) (57) and Oman (64.7%) (58). Despite the increasing level of diabetic cases, advancement in technology and medicine, glycemic level controlling has been insignificant in most diabetic patients in developing countries such as Ethiopia due to glucometer unaffordability and knowledge gaps. Thus, proper patient education and follow-ups are crucial.

As one of the crucial components of self-management for a healthy life, specifically for diabetic patients in our case, physical exercise helps to manage blood glucose levels, minimize cardiovascular risk factors, enhances weight loss, and improves well-being (59). Having this as one of our important points, this study determined the pooled prevalence of poor adherence to recommended physical exercises at 50.45%. This finding disagrees with study reports from Yemen (74.8%) (60) and Ghana (30.67%) (61). This difference might be explained by the fact that the differences in sample size, study settings, and sociodemography have played roles.

In the current study, it has been demonstrated that 24.65% of the Ethiopian diabetic patients were poorly adherent to the prescribed medications. This finding was in-line with study findings from Kenya (28.3%) (62) and a pooled prevalence of poor adherence to anti-diabetic medications in Ethiopia (30.5%) (63). This dimension of self-management behavior was relatively better than other components among Ethiopian diabetic patients but still, it needs more attention and focused approaches.

Furthermore, studies have indicated that social support and self-efficacy are determinant factors of diabetes self-management behaviors (64). Higher social support and self-efficacy Vol. 30, No. 4 July 2020

could improve self-management behaviors among diabetics (65). Thus, these components lead to the improvement in health status but it has been less researched in developing countries like Ethiopia. Therefore, we estimated national pooled prevalence from seven of the 21 included studies, which presented evidence on this component.

The pooled prevalence of poor social support among diabetics in Ethiopia was 54.32%. This finding corroborates with that of another international survey from China (63%) (66), but not with study findings from Malaysia and India (27.3%) (67, 68). The reason for the difference might be that higher income and educational status were observed in the case of studies from elsewhere.

Although our review presented useful information and up-to-date evidence on the prevalence of overall poor self-management and its main components among DM patients in Ethiopia, there were limitations that we mention as follows. Firstly, the overall estimates indicated significant heterogeneity among studies; hence, the interpretation of the finding has to be taken carefully. Whist subgroup analysis and meta-regressions were done we could not specify the heterogeneity source(s). Secondly, due to the lack of published systematic reviews and meta-analysis on poor self-management among diabetic patients, we faced difficulty to compare and contrast with other national pooled estimates.

Apart from the limitations, our work has some strength. For instance, this is the first comprehensive systematic review and metaanalysis so far done on the prevalence of poor self-management behaviors and its main components among diabetic patients in Ethiopia. Hence, it gives strong evidence about the subject matter. Additionally, it also presented most of the self-management components.

The result of this meta-analysis has implications for clinical practice. Diabetes mellitus management could be effective only when diabetic patients adhere to the recommended self-management activities where more than 95% of self-management activities are carried-out by diabetic patients themselves or by their families. Additionally, a high proportion of overall poor self-management and its main components among diabetics is an indicator of poor quality of health care services, specifically diabetes health education. Hence, determining the magnitude of poor self-management has implications to assist Ethiopia's health care system, including the health care providers, to improve the quality of diabetes self-management. In conclusion, the prevalence of poor self-management behaviors among DM patients in Ethiopia is very high. Sizable differences among regions were also observed where the highest and lowest prevalence of poor self-management activities established in Tigray and Addis Ababa regions respectively. Moreover, poor diabetic self-management components were also very high in the country.

The worst levels of poor self-management components were identified in SMBG (80.91%), social support (54.32%), physical exercise (50.45%), and dietary management (49.22%). Whereas relatively better self-management behaviors were observed in foot care (34.32%) and adherence to prescribed medication (24.65%). Thus, our findings indicate that self-management activities among diabetic patients in Ethiopia got the least attention from the patients.

To improve the overall and the main components of self-management behaviors among diabetic patients in Ethiopia, we suggest to all health care workers who involve in DM management to deliver tailored diabetes health education and strict counseling on self-management components. We also recommend to the colleges, universities, ministry of science and higher education, and ministry of health to train and deploy diabetes health educators in all relevant health facilities. Additionally, increasing access to glucometer & its kits, promotion of physical exercises and enhancing social support are strongly suggested to solve the current problems. Furthermore, our findings call for the development and implementation of clearly defined clinical practice guidelines at all levels of health care facilities.
